# A computational approach to understanding effort-based decision-making in depression

**DOI:** 10.1101/2024.06.17.599286

**Published:** 2024-06-19

**Authors:** Vincent Valton, Anahit Mkrtchian, Madeleine Moses-Payne, Alan Gray, Karel Kieslich, Samantha VanUrk, Veronika Samborska, Don Halahakoon, Sanjay G. Manohar, Peter Dayan, Masud Husain, Jonathan P. Roiser

**Affiliations:** 1 Institute of Cognitive Neuroscience, University College London, London, UK; 2 Division of Psychiatry and Max Planck Centre for Computational Psychiatry and Ageing Research, Queen Square Institute of Neurology, University College London, London, UK; 3 Department of Clinical, Educational and Health Psychology, University College London, London, UK; 4 Nuffield Department of Clinical Neurosciences and Department of Experimental Psychology, Oxford University, Oxford, UK; 5 Max Planck Institute for Biological Cybernetics and the University of Tübingen, Tübingen, Germany

**Keywords:** Effort-based decision-making, Depression, Computational Psychiatry, Motivation, Anhedonia

## Abstract

**Background:**

Motivational dysfunction is a core feature of depression, and can have debilitating effects on everyday function. However, it is unclear which disrupted cognitive processes underlie impaired motivation, and whether impairments persist following remission. Decision-making concerning exerting effort to collect rewards offers a promising framework for understanding motivation, especially when examined with computational tools which can offer precise quantification of latent processes.

**Methods:**

Effort-based decision-making was assessed using the Apple Gathering Task, in which participants decide whether to exert effort via a grip-force device to obtain varying levels of reward; effort levels were individually calibrated and varied parametrically. We present a comprehensive computational analysis of decision-making, initially validating our model in healthy volunteers (N=67), before applying it in a case-control study including current (N=41) and remitted (N=46) unmedicated depressed individuals, and healthy volunteers with (N=36) and without (N=57) a family history of depression.

**Results:**

Four fundamental computational mechanisms that drive patterns of effort-based decisions, which replicated across samples, were identified: an overall bias to accept effort challenges; reward sensitivity; and linear and quadratic effort sensitivity. Traditional model-agnostic analyses showed that both depressed groups showed lower willingness to exert effort. In contrast with previous findings, computational analysis revealed that this difference was driven by lower effort acceptance bias, but not altered effort or reward sensitivity.

**Conclusions:**

This work provides insight into the computational mechanisms underlying motivational dysfunction in depression. Lower willingness to exert effort could represent a trait-like factor contributing to symptoms, and might represent a fruitful target for treatment and prevention.

## Introduction

Motivational impairment is common in depression (1–3), relevant to several core symptoms including anhedonia, difficulty making decisions and fatigue, which cluster together and are associated with poorer treatment outcome (4) and lower quality of life (5). Anhedonia, the loss of interest or pleasure in previously enjoyable activities, is a cardinal symptom of depression as well as a transdiagnostic construct observed across multiple disorders (6,7). While anhedonia was originally conceptualised as an inability to experience pleasure (8), several studies have shown that in-the-moment experience of pleasure appears to be preserved in depression (9–14). By contrast, motivational impairments involving decision-making, reward bias and reinforcement learning have repeatedly been shown to be common in depression (15).

A consistent theme emerging from this body of work is that *effort-based decision-making for reward* offers a promising lens through which motivational dysfunction can be understood. This framework proposes a series of cognitive operations associated with cost-benefit decision-making that could potentially underlie motivational symptoms. A ubiquitous finding is that the willingness to engage in effort (physical or mental) depends on both the perceived reward magnitude, and the discounting of that reward by the perceived level of effort (16). While numerous studies have examined the computational and neurobiological mechanisms underpinning effort discounting in healthy individuals (16–21), and contemporary neurocognitive models of depression suggest that lower willingness to exert effort drives depressive symptoms related to motivation (22–24), far fewer studies have investigated these processes in patients experiencing motivational symptoms.

The first systematic attempt to examine effort-based decision-making in depression (i.e., measuring *choices* to exert effort, as opposed to the degree of exertion) was reported by Treadway and colleagues (2012), who compared performance on the *Effort Expenditure for Rewards Task* (EEfRT; (25)) between currently depressed individuals and healthy volunteers. Drawing on an extensive literature in rodents (26), the EEfRT requires participants to choose between a “hard task” (quick button pressing) for more monetary reward, versus an “easy task” (slow button pressing) for less reward, delivered with varying levels of probabilities. Treadway and colleagues reported that depressed participants were less willing to choose the hard task, and less sensitive to both reward and probability. Surprisingly, depressed patients with more severe symptoms were more willing to engage in the hard task; although in item-level analysis, patients with greater reward anticipation dysfunction chose the hard task less. Since this initial study, several other studies have reported comparable results (using the EEfRT or similar tasks) in currently depressed individuals (27–29), although with discrepant results for remitted depression (28,30).

Although these studies provided an important foundation for conceptual advances in our understanding of disrupted motivation in depression, several aspects of their design complicate interpretation. First, most studies, including those examining remitted depression, recruited individuals taking antidepressant medication, which represents a potentially important confound given that antidepressants are known to blunt neural reward processing (31). Second, the level of exertion required to obtain reward was typically not calibrated to individuals; although success rates are generally high on the EEfRT, fast button pressing will inevitably be easier for some participants than others, which is likely to influence the perception of effort. Third, the inclusion of different probability conditions (with no deterministic condition) raises the possibility of an interaction between probability and effort discounting. Fourth, effort levels are not varied parametrically in the EEfRT, and no high-reward/low-effort or low-reward/high-effort options are included, limiting the interpretation of any observed differences. Importantly, individual differences in choices of the high-reward/high-effort option could be driven by sensitivity to either reward or effort.

Another effort-based decision task, the Apple Gathering Task (AGT; (32)), has mainly been used to examine effort processing in neurological conditions (17,33–35), with some studies in schizophrenia (36,37). The AGT differs from the EEfRT in several important ways: a grip-strength device is used for exertion, meaning that the level of force required can be calibrated to each participant; reward and effort are independently parametrically varied; all outcomes are deterministic; and participants either accept or reject offers, as opposed to deciding between high- and low-effort options. Studies using the AGT have reported that patients with Parkinson’s disease (PD; in which depression is very common, (38)) were less willing to exert effort for low rewards (33), especially those with pronounced apathy (34). Other studies reported that experimentally induced inflammation increased sensitivity to effort in healthy volunteers (39), and that relative to matched controls, patients with treatment-resistant schizophrenia were both less willing to exert effort overall and less sensitive to reward (37). However, no study to date has reported behaviour on the AGT in depression.

Over the past decade, computational modelling of behaviour has provided important insights into the cognitive processes underlying disrupted motivation (40). A computational approach provides a systematic analysis of competing, theoretically grounded models of behaviour, by comparing how well each model matches participants’ behaviour. This in turn allows the identification of the latent cognitive processes involved in performing a task. Importantly, computational modelling allows the estimation of specific parameters that distil these processes and govern the behaviour of models, thereby allowing the precise measurement of cognitive processes for each individual. These parameter estimates complement, but also go beyond traditional (model-agnostic) measures of performance, such as proportion of offers accepted, as they provide an explanation of how such measures arise, and also often have superior psychometric properties (41,42).

Therefore, in the present study we performed a computational analysis of performance on the AGT to provide mechanistic insights into the cognitive processes underlying effort-based decision-making in depression. To address the question of whether altered effort-based decision-making is merely a consequence of ongoing depressive symptoms, or if it plays a causal role in their development, we studied four groups, all unmedicated: currently depressed individuals (MDD); individuals remitted from depression (REM); healthy individuals with a depressed close relative, but no personal history (REL - who are known to be at elevated risk); and healthy individuals without any personal or family history of any psychiatric diagnosis (CTR).

Drawing on contemporary neurocognitive models of depression (22–24,43), we hypothesised that lower willingness to exert effort drives depressive symptoms, especially those related to motivation. We therefore predicted that the MDD group would accept fewer offers to exert effort relative to the CTR group, and that this would be driven by lower sensitivity to reward and greater sensitivity to effort. Including the REL and REM groups allowed us to examine whether effort-based decision-making represents a risk factor for depression, and we predicted that a similar pattern of behaviour would be observed in these two high-risk groups. Finally, in exploratory analyses we examined whether specific symptoms such as anhedonia were related to computational parameters.

## Materials and Methods

### Participants

Two studies were conducted, an initial pilot with healthy volunteers (HVs; “Pilot”), and a study including four groups (“Case-control”). In both, participants were compensated £30 for completing symptom questionnaires and a task battery including the AGT (data on other tasks will be reported elsewhere). They could earn a bonus up to £20, depending on performance. All participants were aged 18–60 years, unmedicated and native English speakers. They were recruited through local advertisements, institutional participant databases and local outpatient psychological treatment services, and all provided written informed consent. The study received ethical approval from the UCL Research Ethics Committee (fMRI/2013/005) and the London Queen Square NHS Research Ethics Committee (for depressed individuals: 10/H0716/2).

### Pilot study

One-hundred and two HVs completed the study. Exclusion criteria included any current/past mental health diagnoses (self-report), marijuana use within four weeks, other recreational drug use within one week, or alcohol use within 24hr. The final sample consisted of 67 participants (see Supplemental Information [SI] for exclusions).

### Case-control study

Sixty-two HVs (CTR), 38 HVs with a depressed first-degree relative (REL), 50 remitted depressed participants (REM), and 51 currently depressed participants (MDD) completed the study. Diagnoses were assessed using the Mini International Neuropsychiatric Interview v5 (MINI; (44)). Family history of mental illness was assessed using the Family Interview for Genetic Studies (FIGS; (45,46)).

MDD participants had to meet criteria for a current major depressive episode (MDE) according to the MINI, with a score≥8 on the Hamilton Depression Rating Scale (HAM-D; (47)); REM participants had to meet criteria for a past MDE, with a score≤7 on the HAM-D. REL and CTR participants had no current or past diagnoses, with a score≤7 on the HAM-D, and REL participants had at least one first-degree relative with current/past depression.

Additional exclusion criteria for all groups included: any history of neurological disorders; current use of psychotropic medication (including St John’s Wort) within the past four weeks (eight weeks for fluoxetine); marijuana use within the past four weeks or other recreational drug use within the past week; alcohol use within the past 24hr; lifetime manic/hypomanic episode; current alcohol/substance use disorder; lifetime severe alcohol/substance use disorder (occurring outside a depressive episode); and lifetime history of any psychotic disorder. Current or past anxiety disorders and severe alcohol/substance use disorder occurring exclusively within a depressive episode were not exclusion criteria for the MDD/REM groups.

The final sample included 57 CTR, 36 REL, 46 REM and 41 MDD participants (see SI for exclusions). With this sample size, at alpha=0.05, we had 80% power to detect effect sizes of at least f=0.25 (medium effect size).

### Experimental procedure

For the Pilot study eligibility was assessed using a structured telephone interview. For the Case-control study, participants attended a screening session completing the MINI, FIGS and questionnaires before returning for cognitive testing on a separate day.

In both studies, participants completed the digit span forwards and backwards (WAIS-III; (48)), the Wechsler Test of Adult Reading (WTAR; (49)), the Apathy Evaluation Scale (AES; (50)), the Beck Depression Inventory (BDI-II; (51)), the Dysfunctional Attitudes Scale (DAS-SF1–2; (10)), the Life Orientation Test-Revisited (LOTR; (52)), the Snaith-Hamilton Pleasure Scale (SHAPS; (53)), the State-Trait Anxiety Inventory for Adults (STAI; (54)), and the Temporal Experience of Pleasure Scale (TEPS; (55)). In the Pilot study, participants completed the Chapman Physical Anhedonia Scale (CPAS; (56)). In the Case-control study, participants completed the HAM-D.

### Apple Gathering Task (AGT)

The AGT measures willingness to exert physical effort for reward (33). Before the main task, participants performed a six-trial calibration phase, squeezing a hand-dynamometer as hard as they could with their non-dominant hand to fill an on-screen gauge. The peak force from the last three trials determined their MVC (maximum voluntary contraction), setting comparable effort levels across subjects. Participants then attempted four effort levels (20%, 40%, 60% and 80% of MVC).

**Figure 1:**
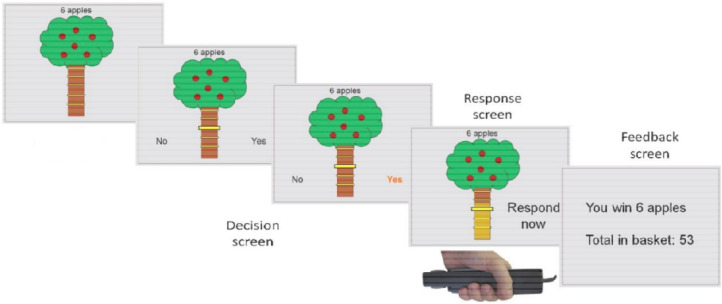
Apple Gathering Task (AGT). On each trial, participants are given a different offer comprising a number of apples (3, 6, 9, or 12 apples) for a given effort cost (20%, 40%, 60% or 80% of their maximum grip strength). Participants can either accept the offer or refuse the offer. If the offer is accepted, participants need to squeeze the gripper to the required effort level (or above) for 3 seconds in order to win the apples on this trial.

On each trial in the main task, participants saw a tree containing a number of apples representing the available reward, and a bar on the tree trunk representing the force required to obtain the apples (Figure 1; see SI). There were four levels of reward (3, 6, 9, or 12 apples) and effort (20%, 40%, 60% and 80%). Each reward/effort combination was repeated five times, resulting in a total of 80 trials. Participants could accept or refuse offers. For refused offers, ‘no response required’ was displayed, followed by the next decision. An accepted offer required squeezing the hand-dynamometer at or above the effort level to win points, followed by feedback. To mitigate fatigue, the exertion phase was omitted and “No response required” appeared on 25% of accepted trials.

### Statistical Analysis

Repeated-measures analysis of variance (ANOVA) was used to examine whether acceptance rates varied as a function of reward, effort, or their interaction. For the Case-control study, group was added as a between-subjects measure. Greenhouse-Geisser correction was applied when sphericity violations occurred. Acceptance rates were arcsine transformed prior to analysis to satisfy Gaussian assumptions. Decision reaction times and success rates were also analysed (see SI). Covariates in analyses (age, see below) were mean corrected.

Questionnaire data was synthesized using exploratory factor analysis on the total score of each questionnaire using Promax rotation (see SI).

### Computational Analysis

Computational analysis was performed to compare competing hypotheses of reward and effort contributions to decision-making (model comparison and selection), and to estimate participant-level parameters corresponding to reward and effort processing (parameter estimation). All models were implemented using hierarchical Bayesian estimation in Stan (57) with parameters estimated using Hamiltonian Markov-Chain Monte Carlo sampling. This approach assumes that each group of participants can be described using a population distribution, which improves parameter estimation accuracy, and includes priors over the parameters of this distribution, which acts as soft constraints on likely parameter ranges.

Participants in the Pilot study were fit under the same group-level priors. For the Case-control study, all participants were fit under the same prior for parameters of no interest and separate group-level priors were used for the parameter of interest (one parameter at a time; see SI). Sensitivity analyses with different group priors did not materially impact the estimated parameters or group comparison results (Figure S2).

### Winning model

Seventy models with varying complexity were compared to select the winning model (which best captured decisions, whilst accounting for model complexity; see SI). For brevity we only present the winning model in the main text (Figure S1; see SI). This model comprised four parameters: two effort sensitivity terms (linear: LinE, and quadratic: E^2^), a linear reward sensitivity term (LinR), and an acceptance intercept/bias term (K). Together these parameters captured the full range of choice patterns observed across participants.

The model operates as follows: for each offer, the effort level is transformed through the effort sensitivity parameters to yield a subjective value of effort (eq.1). The linear effort sensitivity term scales the value of the effort level (more negative=greater subjective effort), and the quadratic term allows the effort profile to either taper off (effort sensitivity^2^>0), or to increase disproportionately with increasing effort (effort^2^ sensitivity<0).


eq. 1 $$Subjective\;value\;of\;effort=(LinE\times \;effort)+\left(E^{2}\times \right.\;\left.{effort}^{2}\right)$$

Reward is transformed through a linear reward sensitivity term to yield a subjective value of reward (eq.2) (more positive=greater subjective reward).


eq. 2 Subjectivevalueofreward=(LinR×reward)

The subjective values of reward and effort are then combined to form the subjective value of the offer (eq.3).


eq. 3 Subjectivevalueofoffer=Subjectivevalueofreward+Subjectivevalueofeffort

The subjective value of the offer is passed through an inverted logit link function with a bias parameter (eq.4). This maps the subjective value of the offer to a probability of acceptance, where the bias (or intercept) term shifts the curve by a constant. The bias term therefore represents the overall tendency to accept offers (higher=more likely to accept), independent of reward or effort.


eq.4. Acceptprobability=inv_logit(K+Subjectivevalueofoffer)

Posterior predictive checks suggested that the model could accurately recapitulate both the group-level pattern of responding and individual differences in acceptance rates (Figure S3; Pilot: r=0.997; Case-control: r=0.999). All parameters showed high recoverability (Figure S4). The LinE term was included as a constant term in the Case-control study, but was a free parameter in the Pilot study (see Results).

## Results

Participant characteristics are presented in Table 1. The Case-control groups did not differ significantly on any variable.

### Model-agnostic AGT analysis

**Figure 2:**
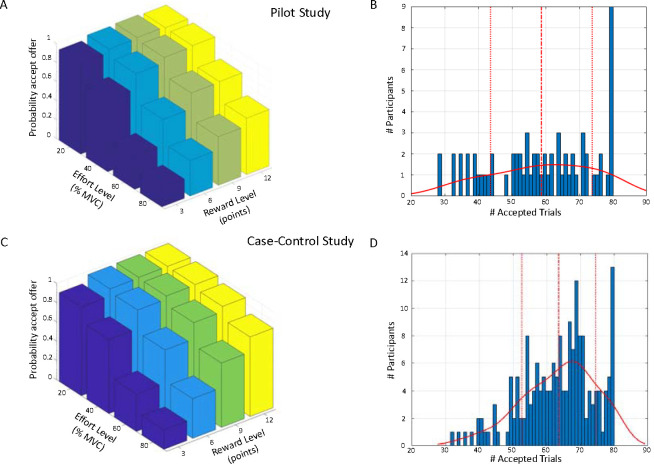
Acceptance rates. (A) Average acceptance rates as a function of reward level (number of points) and effort level (% MVC) for the Pilot study. (B) Distribution of the number of accepted offers (out of 80) in the Pilot. (C) Average acceptance rates as a function of reward level and effort level across all groups in the Case-control study. (D) Distribution of the number of accepted offers (out of 80) across all groups in the Case-control study. Continuous lines denote the kernel density estimate from the data, dashed lines denote the mean, and dotted lines represent ± 1 standard deviation from the mean.

In initial analyses we determined that older individuals accepted more offers overall (Pearson r=0.20, p=0.014), and therefore age was included as a covariate in all Case-control analyses. We observed wide variability in overall acceptance rates, ranging from 35–100% in the pilot study, and 40–100% in the Case-control study (Figure 2B&D). As expected, acceptance rates decreased significantly as effort increased (Pilot: F(1.57, 103.77)=120.19, p<0.001; Case-control: F(1.75, 306.31)=351.79, p<0.001), and increased significantly as reward increased (Pilot: F(1.63, 107.57)=92.02, p<0.001; Case-control: F(1.65, 288.06)= 325.45, p<0.001). There was a significant reward-by-effort interaction for both studies, driven by particularly high acceptance rates (at ceiling) for high-reward/low-effort trials (Pilot: F(4.25, 280.88)=16.42, p<0.001; Case-control: F(4.95, 866.79)= 90.13, p<0.001; Figure 2A&C).

**Figure 3:**
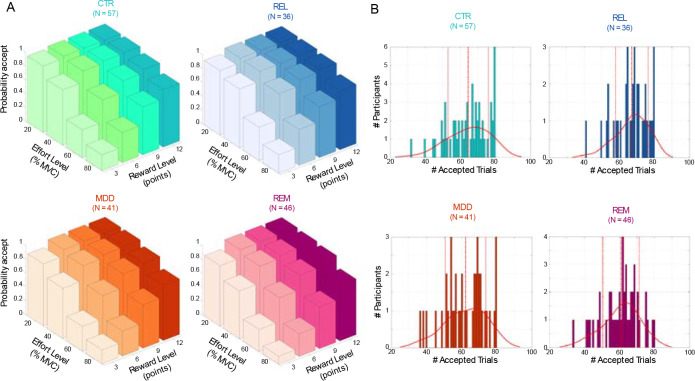
Acceptance rates for the Case-control study. (A) Average acceptance rate as a function of reward level (points) and effort level (% MVC) for the control (CTR), first degree relatives (REL), patients with current depression (MDD), and remitted depression (REM) group. (B) Distribution of the number of accepted offers for each group. Continuous lines denote the kernel density estimate from the data, dashed lines denote the mean, and dotted lines represent ± 1 standard deviation from the mean.

In the Case-control study we found a significant main effect of group (F(3,175)=3.26, p=0.023; Figure 3), but all interactions with group were non-significant. Post-hoc tests revealed significantly lower acceptance rates for the REM and MDD groups compared to REL (REM vs REL: Cohen’s d=0.63, p=0.007; MDD vs REL: Cohen’s d=0.41, p=0.015; effect sizes here and below do not include covariates).

There was no significant group difference in either success rates or decision RTs (all p>0.05). Importantly, success rate at the highest effort level was above 80% across all groups (Figure S5).

### Questionnaire factor analysis

Factor analysis was performed on questionnaire measures for both studies. Despite minor differences in measures (CPAS was only included in the Pilot study; HAM-D was included only in the Case-control study), a similar four-factor solution was obtained in both studies (Table 2). We named each factor based on the scales displaying the highest loadings (>0.3). The “Low-mood” factor included high loadings for BDI-II, HAM-D, LOTR and STAI. An “Apathy” factor mostly comprised the AES sub-scales, and to a lesser extent SHAPS and STAI-state in the Case-control study. A “Hedonia” factor included the SHAPS and TEPS (NB due to scoring conventions these load with opposite signs), and the CPAS (in the Pilot study). Finally, the two DAS sub-scales loaded almost exclusively onto their own “Dysfunctional Attitudes” factor.

### Computational AGT analysis

#### Model comparison

**Figure 4:**
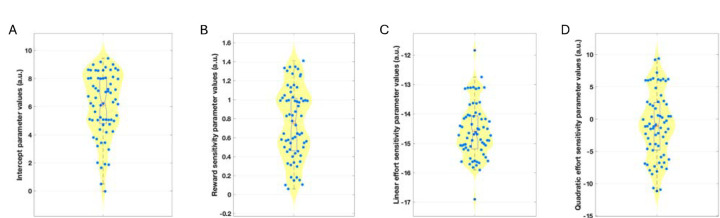
Estimated model parameters for the Pilot study. Figures are showing violin and boxplots as well as the mean (plus sign) and median (notch) for (A) estimated intercept/bias (K), (B) reward sensitivity (LinR), (C) linear effort (LinE), and (D) quadratic effort sensitivity (E^2^) parameter values from the winning model.

The winning model in both studies was a derivative of a four-parameter model including an intercept (bias) parameter (K), linear reward (LinR) and effort (LinE) sensitivity parameters, and a quadratic effort parameter (E^2^; Figure 4; Figures S6&7). In the Case-control study, owing to high trade-off between the LinE and E^2^ parameters and limited LinE range, the LinE term was constrained to that of the average LinE parameter estimated from the Pilot study (ConstE=−15; see SI).

#### Case-control comparisons

**Figure 5:**
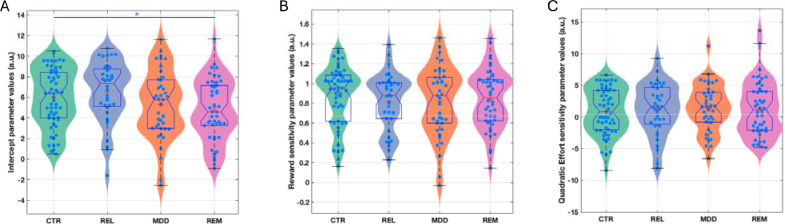
Estimated model parameters for the Case-control study. Figures show violin and boxplots as well as the mean (plus sign) and median (notch) for (A) estimated intercept/bias (K), (B) reward sensitivity (LinR), and (C) quadratic effort (E^2^) sensitivity parameter values from the winning model. CTR: Control group; REL: First-degree relative group, MDD: Current depression group; REM: Remitted depression group. *Denotes significance at p<0.05.

Participant-level parameters (K, LinR, E^2^) were compared between the groups including age as a covariate (Figure 5). The K (acceptance bias) parameter differed significantly between the groups (F(3,175)=3.19, p=0.025). Post-hoc tests revealed that this was driven by the REM (Cohen’s d=0.61, p=0.006) and MDD (Cohen’s d=0.41, p=0.032) groups having a lower acceptance bias than the REL group and the REM group having a lower acceptance bias than the HC group (Cohen’s d=0.41, p=0.04). Planned comparisons revealed that the combined REM+DEP group had a lower intercept parameter than the combined REL+CTR group (Cohen’s d=0.39, F(1,177)=8.26, p=0.005). However, the combined REL+REM+DEP group did not differ from the CTR group (Cohen’s d=0.16, p=0.276). No other parameter differed significantly between groups (LinR: F(3,175)=0.33, p=0.806; E^2^: F(3,175)=0.13, p=0.942). This suggests that lower acceptance rates in the depression groups were driven by a lower acceptance bias and not alterations in reward or effort sensitivity.

### Associations between parameters and symptoms

#### Pilot study

**Figure 6:**
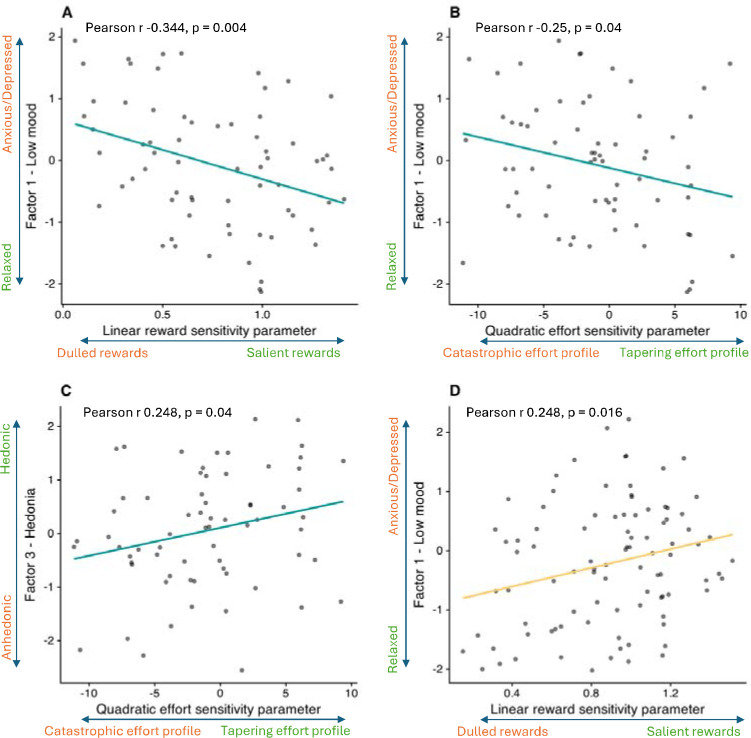
Correlations between computational parameters and symptom factors. (A) Correlation between the Low Mood factor and the linear reward sensitivity (LinR) parameter in the pilot study. (B) Correlation between the Low Mood factor and the quadratic effort sensitivity (E^2^) parameter in the pilot study. (C) Correlation between the Hedonia factor and the quadratic effort sensitivity (E^2^) parameter in the pilot study. (D) Correlation between the Low Mood factor and the linear reward sensitivity (LinR) parameter in the case-control study for the CTR+REL group only.

In the Pilot study, LinR correlated negatively with the Low Mood factor (r=−0.344, p=0.004; Figure 6), suggesting that participants with greater anxiety/depression subjectively perceived rewards as less valuable. The E^2^ parameter correlated negatively with the Low Mood factor (r=−0.25, p=0.04) and positively with the Hedonia factor (r=0.248, p=0.043), suggesting that participants with greater anxiety/depression and anhedonia perceived higher levels of effort as disproportionately costly.

#### Case-control study

In the Case-control study, however, no significant associations between symptom factors and computational parameters were observed in either the combined CTR+REL+REM sample or in the MDD group alone. In the combined CTR+REL group, surprisingly (and in contrast to the corresponding result in the Pilot study) LinR correlated positively with the Low Mood factor (r=0.248, p=0.016; Figure 6).

## Discussion

Impaired motivation is a hallmark of depression, but the cognitive and computational processes that drive motivational symptoms are poorly understood. We confirm that motivation can be captured effectively within an effort-benefit decision-making framework and dissected into specific cognitive components using a computational approach. Consistent with our hypotheses, we found that depression is characterised by a lower willingness to engage in effort. Our computational analyses suggest that this difference was not driven by altered sensitivity to reward or effort, but rather by a lower overall inclination to exert effort, reflected in the acceptance bias parameter. The presence of lower acceptance bias in both the remitted and currently depressed groups suggests that this attribute of decision-making could represent a trait-like feature, as proposed by neurocognitive models of depression (22–24,43), or possibly a “scar” effect of having previously experienced depression (58).

Our findings are consistent with previous work demonstrating lower willingness to exert effort for reward in depression (25,27–29,59). However, a key assumption of prior work has been that low motivation is driven by mis-calibrated valuation of either reward or effort (21,60,61). By carefully examining a large space of computational models, we provide evidence against this assumption, and suggest that a different process is at play: effort acceptance bias. While surprising and not entirely straightforward to interpret, this result clarifies prior findings from tasks that were unable to disambiguate between these factors. One possibility is that lower acceptance bias could be driven by lower confidence in being able to achieve the required effort, despite the individual calibration of required exertion. This would indicate that metacognitive processes might play a role in motivational impairment. Indeed, previous studies have identified that transdiagnostic symptoms of apathy, poor self-esteem and low mood are associated with low overall confidence in perceptual decisions (62,63), independent of accuracy, mirroring our findings. This could plausibly be related to low global expectations of success and fear of failure in depression (64–66).

An important goal of neuroscientific research in depression is to determine whether cognitive disruptions are simply a consequence of depressive symptoms, or whether they contribute causally to their development (67). We observed a similar pattern of effort-based decisions for reward in both current and remitted depressed groups. This suggests that a general bias against exerting effort might represent a core feature of depression, not simply an epiphenomenon driven by symptoms. However, we did not observe a similar pattern in never-depressed first-degree relatives. Therefore, an alternative interpretation might be that lower willingness to exert effort is a consequence rather than a cause of depression, which does not recover after symptoms remit (akin to a ‘scar’ effect). A third possibility is that the first-degree relatives we tested were not at elevated risk for depression, but were instead actually resilient. Depression often presents early in life and our REL sample was older (mean age~26) than the peak onset of depressive symptoms (68,69). Thus, many individuals in this group might never go on to develop depression, having passed a period of high risk. Future studies should recruit younger samples and test these predictions longitudinally.

If confirmed as a true risk factor for depression, lower overall willingness to exert effort may represent a fruitful target for intervention. Such targets are sorely needed for motivational symptoms, which are particularly difficult to treat and constitute an area of unmet clinical need (4,70). Treatments that can boost engagement in activities through altering this computational bias could be particularly effective in treating or even preventing depression. Elements of behavioural activation (BA) therapy, such as activity scheduling, might play such a role, as these rely on acting on pre-planned activities rather than internal states (71). Interestingly, this BA component has recently been shown to alter effort processing in healthy participants, offering proof-of-concept of this idea (72).

### Limitations

While our model of effort-based decision-making replicated across studies, the associations with specific facets of motivational symptoms were inconsistent. One possible reason might be that our Case-control sample size was too low to allow associations to be replicated, especially the MDD group. Future studies should explore these associations further in larger samples to better detect associations.

An important strength of the effort task we employed is its individually tailored effort levels, which resulted in >80% average success rates at all effort levels. Nonetheless, it remains possible that differential success rates across effort levels could have influenced decisions. Importantly, however, there was no group difference in success rates—or in effort sensitivity—making it unlikely that the lower effort acceptance bias we observed in depression was influenced by success rates.

### Conclusion

This study advances our understanding of motivational impairment in depression by identifying effort acceptance bias, independent of reward or effort sensitivity, as a computational feature associated with both current and remitted depression. These results raise the possibility that interventions that boost this bias may improve the effectiveness of treatment. Taking a computational perspective to understanding effort-based decision-making offers a promising avenue to understand the multifaceted nature of motivational impairment in depression.

## Supplementary Material

Supplement 1

## Figures and Tables

**Table 1:** Participant characteristics.

	Pilot			Case-Control		

Sample	HV (N=67)	CTR (N=57)	REL (N=36)	REM (N=46)	MDD (N=41)	Statistics (Case-control)

Age	*28.45 (9.88)*	*26.70 (8.14)*	*26.06 (8.19)*	*26.91 (7.06)*	*30.24 (11.57)*	*F(3,176)=1.87, p>0.05*
Gender (M/F)	*23/44*	*18/39*	*13/23*	*17/29*	*12/29*	*X(3)=0.78, p>0.05*
Digit Span forward	*9.86 (1.90)*	*9.35 (1.86)*	*9.28 (1.97)*	*9.41 (1.85)*	*9.07 (2.08)*	*F(3,176)=0.26, p>0.05*
Digit Span backward	*8.20 (2.36)*	*7.84 (2.23)*	*7.06 (2.27)*	*8.04 (2.28)*	*7.44 (2.31)*	*F(3, 176)=1.54, p>0.05*
IQ (WTAR)	*109.79 (11.47)*	*111.91 (7.43)*	*113.15 (7.65)*	*116.14 (8.08)*	*114.51 (9.95)*	*F(3,172)=2.33, p>0.05*
Years Education	--	*16.51 (2.82)*	*17.19 (2.97)*	*17.04 (2.82)*	*15.68 (2.31)*	*F(3, 176)=2.51, p>0.05*
HAM-D	--	*0.58 (1.05)*	*1.06 (1.35)*	*1.35 (1.98)*	*17.00 (5.40)*	--
BDI-II	*3.39 (4.40)*	*2.09 (2.71)*	*2.78 (4.08)*	*5.00 (5.22)*	*28.78 (7.95)*	--
STAI Trait	*34.96 (9.18)*	*32.88 (7.58)*	*34.86 (11.63)*	*39.07 (9.50)*	*63.46 (8.10)*	--
SHAPS	*24.34 (5.98)*	*21.93 (5.39)*	*21.75 (4.75)*	*23.30 (4.59)*	*37.20 (5.52)*	--
TEPS-A	*46.99 (7.00)*	*44.83 (7.11)*	*46.03 (7.85)*	*43.63 (6.69)*	*30.05 (8.49)*	--
TEPS-C	*36.60 (6.79)*	*36.04 (5.95)*	*37.78 (6.16)*	*37.30 (6.85)*	*25.90 (7.46)*	--

HV: Healthy Volunteers (in the Pilot study); CTR: Control Participants; REL: Participants with at a first-degree relative with Depression; REM: Remitted depressed participants; MDD; Currently depressed participants; WTAR: Wechsler Test of Adult Reading; HAM-D: Hamilton Depression Rating Scale; BDI-II: Beck Depression Inventory; STAI: State Trait Anxiety Inventory; SHAPS: Snaith Hamilton Pleasure Scale; TEPS-A: Temporal Experience of Pleasure Scale - Anticipatory subscale; TEPS-C: Temporal Experience of Pleasure Scale - Consummatory. Brackets represent standard deviations. In the Pilot study, there was missing data for the digit span forward (N=2), digit span backward (N=3) and IQ (N=4). IQ data were missing for four Case-control participants (REM N=2, REL N=2).

**Table 2:** Factor analysis solutions for the Pilot and Case-control (excluding the MDD group) study questionnaire measures.

	Pilot				Case-Control			

*Estimated Factors*	LOW MOOD	APATHY	HEDONIA	DYSFUNC. ATTITUDES	LOW MOOD	APATHY	HEDONIA	DYSFUNC. ATTITUDES
	FACTOR 1	FACTOR 2	FACTOR 3	FACTOR 4	FACTOR 2	FACTOR 1	FACTOR 3	FACTOR 4
	
*HAM-D*	--	--	--	--	**0.60**	−0.01	0.00	−0.20
*BDI-II*	**0.51**	−0.05	−0.34	−0.03	**0.57**	0.15	0.12	0.13
*AES Cognitive Apathy*	−0.06	**0.84**	−0.11	0.00	0.01	**0.76**	−0.07	−0.11
*AES Behavioural Apathy*	0.25	**0.66**	0.15	−0.09	0.15	**0.67**	0.04	−0.02
*AES Emotional Apathy*	−0.24	**0.59**	−0.21	−0.02	−0.19	**0.66**	−0.11	0.04
*AES Other Apathy*	0.00	**0.83**	0.15	0.09	0.03	**0.87**	0.30	−0.02
*Chapman Physical Anhedonia*	−0.20	0.07	**−0.87**	0.02	--	--	--	--
*DAS Perfectionism*	−0.06	0.03	−0.04	**1.01**	−0.01	−0.08	0.04	**1.05**
*DAS Social Approval*	0.08	0.01	0.02	**0.61**	0.28	0.03	0.16	**0.36**
*LOTR Optimism*	**−0.47**	−0.08	0.22	−0.18	**−0.50**	0.00	0.25	−0.05
*SHAPS*	0.25	0.01	**−0.33**	−0.16	−0.05	**0.39**	**−0.31**	0.07
*STAI State*	**0.78**	0.03	0.13	0.02	**0.56**	**0.30**	−0.01	−0.03
*STAI Trait*	**1.11**	−0.04	0.14	−0.05	**0.96**	−0.06	−0.09	0.00
*TEPS-A.*	−0.04	−0.23	**0.25**	0.03	−0.08	0.17	**1.04**	0.06
*TEPS-C.*	0.06	0.09	**0.90**	−0.03	0.02	−0.08	**0.42**	−0.18

Questionnaire loadings >0.3 (or highest loading factor) are highlighted in bold. A consistent factor solution was identified between the two studies, other than the order of the first two factors. HAM-D: Hamilton Depression Rating Scale; BDI-II: Beck Depression Inventory; AES: Apathy Evaluation Scale; DAS: Dysfunctional Attitudes Scale; LOTR: Life Orientation Test-Revised; SHAPS: Snaith Hamilton Pleasure Scale; STAI: State Trait Anxiety Inventory; TEPS-A: Temporal Experience of Pleasure Scale - Anticipatory subscale; TEPS-C: Temporal Experience of Pleasure Scale - Consummatory.
